# A bibliometric review on latent topics and research trends in the growth mindset literature for mathematics education

**DOI:** 10.3389/fpsyg.2022.1039761

**Published:** 2022-11-29

**Authors:** Xiaoyu Xu, Qiaoping Zhang, Jin Sun, Yicheng Wei

**Affiliations:** ^1^Department of Mathematics and Information Technology, The Education University of Hong Kong, Hong Kong, Hong Kong SAR, China; ^2^Department of Early Childhood Education, The Education University of Hong Kong, Hong Kong, Hong Kong SAR, China

**Keywords:** growth mindset, mathematics education, bibliometric analysis, student learning, mathematics learning, bibliometric review

## Abstract

Embracing a growth mindset is essential to students’ academic improvement. This manuscript aims to better understand the existing literature on the role and effects of the growth mindset in mathematics teaching and learning. It provides an updated perspective on the research regarding the growth mindset in mathematics education. The dataset comprises 85 journal articles published from 2012 to 2022 retrieved from the Web of Science (WOS) and Scopus databases. The current study applies a methodology based on bibliometric analysis techniques. The analysis reveals and corroborates several patterns from the research trends, journals, countries, and authors that have significant impacts on the research field. The findings show that USA, UK, and Norway are the most productive countries in publishing research on the topic. Moreover, the results of the thematic analysis indicate that the topics discussed among most of the articles in the dataset include engagement, implementation, persistence, children, fluid intelligence, and skills. The longitudinal trends in research themes based on study keywords illustrate an evolution in the research from the concept of mindsets to implicit theories on the growth mindset alongside academic achievement. Lastly, this study also provides an overview of the conceptual structure underlying studies on the growth mindset, which offers valuable insights into potential research topics for academics and practitioners seeking to explore the growth mindset in the future.

## Introduction

Entering the early 21st century, we have witnessed lots of changes and evolvements. Amid globalization, the growth of information and communication technology (ICT), and knowledge sharing, the content of education is changing (Trilling and Fadel, 2009; Voogt and Roblin, 2012). To cope with our ever-changing society, education should equip students with essential skills to enable them to thrive and succeed in their future. In 2018, the Organisation for Economic Co-operation and Development [OECD] (2018) proposed the Learning Compass 2030 framework, emphasizing the importance of critical thinking, meta-cognitive skills, learning-to-learn skills, and the ability to learn attitudes and values.

In education, students’ mindset strongly influences their learning performance (Dweck, 2017a). A student’s mindset refers to the attitudes, beliefs, and expectations they have about a course or subject (Chew, 2014; Dweck, 2017b). These types of mindsets can be a boon or a barrier to learning. Students with a fixed mindset tend to believe intelligence is fixed, and that they are born with a particular set of skills and cannot change them (Dweck, 2006). Children with a fixed mindset are concerned with how they will be judged, and they want to make sure they succeed (Dweck, 2017b). In contrast, students with a growth mindset see intellectual ability as a malleable trait that could be cultivated and enhanced through personal effort and guidance (Dweck, 2015). They are concerned with improving. For these children, success is about stretching themselves (Dweck, 2017b). These students are more likely to embrace intellectual challenges as opportunities to learn and grow, and to be more resilient in the face of setbacks (Yeager and Dweck, 2020). The National Council of Teachers of Mathematics (2014) reported that “believing in, and acting on, growth mindsets versus fixed mindsets can make an enormous difference in what students accomplish” (p. 64). When it comes to mathematics learning, mindset is of particular importance. Students with mathematical problem-solving and critical thinking skills are among the strongest performers with a growth mindset in overall mathematical achievement (Organisation for Economic Co-operation and Development [OECD], 2013). To nurture a growth mindset for students, we should also examine teachers. Studies have revealed that teacher mindsets can influence students’ mindset and directly affect their achievement (Ostroff, 2016; Ronkainen et al., 2019). Kamins and Dweck (1999) observed that when teachers believed in their students’ ability in achieving success, the students could stretch their limits and exceed expectations. However, in this research area, many empirical studies have focused on students, and more research needs to be conducted on how to develop a growth mindset in teachers (Guidera, 2014).

Although positive correlations or influences have been found between students’ growth mindset and their performance (Dweck, 2017a), to what extent and in what kinds of learning areas these effects hold are unclear. A more holistic analysis of specific empirical studies is required. Notably, most studies lack a comprehensive understanding of the entire growth mindset in mathematics education. For example, whether a growth mindset approach exists for other learning and teaching topics in mathematics education remains unanswered. Furthermore, to the best of our knowledge, no previous efforts have been made to conduct a bibliometric review of the literature in this field, a widely used mathematical and statistical tool for quantitative research (Pritchard, 1969; Chen et al., 2018). Thus, this article aims to fill this gap by systematically reviewing the literature with the bibliometric method, and to summarize current literature findings. We examine empirical studies on the growth mindset in mathematics education over the last 10 years and provide a more detailed picture of latent topics, development trends, collaborative organizations, and annual topic distributions. This study also further discusses the representative research work and also suggests a possible pathway for future research.

The current review investigates the following research questions:

(1) Which countries/regions were major contributors to growth mindset research in the last decade?
(2) What were the primary research topics for the growth mindset in mathematics education and their significance to our society (i.e., students, teachers, school, broader society)?
(3) How did research topics evolve through the years?
(4) What could be the possible research directions in the future?

## Literature review

To better understand the development of the growth mindset in mathematics education, we first briefly introduce some key concepts in growth mindset research. This section analyzes three aspects, including the definition of the growth mindset, interventions for the growth mindset, and the growth mindset in mathematics education, to review and describe state-of-the-art research in mathematics education.

### Definition of the growth mindset

Mindset can be understood as the influence of past thinking on current thinking. It is a collection of beliefs related to continual learning and the malleability of intelligence (Dweck, 2006). Mindset could be classified into two types: the fixed mindset and the growth mindset (Dweck et al., 1995; Dweck, 2006). A person with a fixed mindset believes that intelligence is a stable, unchangeable trait. Conversely, a person with a growth mindset believes that intelligent skills could be cultivated and developed through effort.

Previous studies have interpreted the effect of different mindsets on student learning and teachers’ teaching, especially when they struggled with problems or failure. Students with a fixed mindset tend to avoid challenges, quit when they encounter challenges, and ultimately achieve less academic success (Dweck, 2006; Smiley et al., 2016). Conversely, when students learn with a growth mindset, they can improve with effort and guidance. They are more willing to accept challenging work and persevere through obstacles by exploring new tactics or increasing their efforts. Those students realize and appreciate the importance of trial and error, where they can learn from mistakes and alter their tactics (Dweck, 2006; Boaler, 2015). A mindset might change with different contexts and over time. Teachers’ understanding and explanation of mindset theory could help students change their mindset toward learning mathematics and promote their positive beliefs and attitudes toward the subject (Boaler, 2015).

### Interventions for promoting the growth mindset

Replicated studies (Sisk et al., 2018; Yeager et al., 2019) show that mindset treatments have a positive impact on student learning achievements. Hence, changing students’ mindset from a fixed type to a growth type becomes crucial. Yeager and Dweck (2020) identified instructional interventions that assist struggling students in tracking their progress. By adopting a specific program or a series of steps to target an academic need, these interventions were expected to help kids with learning troubles in subjects such as mathematics. Moreover, Yeager and Dweck (2020) proposed that any intervention should describe actionable steps for developing a growth mindset. For instance, individuals can train their brains by attempting challenging schoolwork. They may also benefit from hearing about notable people or colleagues with a development mindset. Nevertheless, interventions should not be passive actions; they should require individual reflection. For instance, as part of an intervention program, students may compose a brief essay about how they have developed their abilities through challenges and how they want to adopt a growth mindset in their future endeavors. Students may also compose a letter or write what they would communicate to their peers; this exercise can determine which students have a fixed mindset. Ultimately, interventions should not merely highlight the effort but also show that learning abilities have the potential for improvement. This does not mean that learning abilities can be readily altered or considerably modified, but that the potential for change exists (Yeager and Dweck, 2020). Vongkulluksn et al. (2021) mentioned that the learning process should be highlighted rather than the results of learning. Students should learn to acquire and generalize strategies and resources that they could apply in future work. Teachers could play their part in helping students to go through failure or setbacks and appreciate them as part of the learning process. Failure would offer crucial feedback on improvements and help build knowledge (Dweck, 2017a). Feedback is vital and should be matched with the learning objectives that students are aiming to achieve.

### The growth mindset in mathematics education

Having a growth mindset would help students understand that they could improve their mathematical abilities with effort. Holding a growth mindset in mathematics learning meant that students could leverage a particular thinking procedure to solve mathematical problems and were willing to attempt the task various times despite setbacks. This kind of mindset would gradually transform into a habitual response (Dweck, 2008). Solving problems multiple times through trial and error cultivated a growth mindset, which helped students learn mathematics and strengthened their belief in the possibility of growth in their intelligence (Dweck, 2015). Students with a growth mindset believed that the more they learnt, ranging from mathematical principles to calculation methods, the better their mathematical thinking skills, driving a virtuous cycle in their continuous learning (Ng, 2018). They understood that their objectives in learning mathematics were to think, understand, and grow. However, when students treated mathematical problems as just a series of short questions, they could not appreciate their own cognitive development in small steps and the wider applications of learning mathematics (Boaler, 2015). They perceived that there were only fixed methods for solving particular mathematical problems.

Several studies on students’ mathematics learning attitudes considered the students’ mindset to be an important factor in developing their problem-solving skills. The present study addresses the growth mindset in mathematics learning. The growth mindset in mathematics learning refers to how an individual thinks while learning mathematics, reflecting their number sense, logical thinking ability, judgment ability, and speculative ability (Hakim and Nurlaelah, 2018). A growth mindset highlighting the learning process was significant for developing students’ problem-solving skill, and beneficial to their continuous pursuit of learning (Dweck, 2006; Boaler, 2015). People with a growth mindset in mathematics learning believed that their mathematical abilities could be developed through learning and training, and their intelligence was malleable rather than fixed.

Turning to other studies, Daly et al. (2019) proposed that students’ mindsets could produce either positive or negative effects on their mathematics learning. The positive effects included students building certain mathematical thinking patterns which could be applied to solving new problems. Therefore, when conditions remained unchanged, the existing thinking patterns could help students quickly process the numbers and formulas, and then associate and mobilize their learned knowledge and skills to quickly respond to the environment. A positive effect enabled people to quickly extract familiar information from the original cognitive structure and choose the correct direction of thinking, thus contributing to the development of new knowledge. In Eichhorn’s (2016) study on Indian primary students’ number sense, it was found that, to some extent, the students’ negative mindset will limit the divergence of their thinking, making it difficult for them to think flexibly in new environments, leading them to be easily influenced by their old thinking. In this sense, a growth mindset should be even more crucial for helping students change their current way of thinking.

Lastly, the growth mindset also has important implications for the development of subjective task values, including intrinsic value, utility value, and attainment value. The growth mindset places greater emphasis on mastery-oriented or learning goals, while the fixed mindset prefers to endorse performance goals (Blackwell et al., 2007; Burnette et al., 2013). Learning goals emphasize the importance of improving individual ability and expanding skill sets. In contrast, performance goals emphasize the importance of demonstrating a high ability (performance approach) and avoiding the external perception of low ability (performance-avoidance) (Elliot and Harackiewicz, 1996). When facing a challenging task, individuals with fixed mindsets would worry about their own incompetency in performing the task, which in turn undermines their intrinsic interest or enjoyment during the process (Dweck, 2008; Stipek and Gralinski, 1996). To conclude, the growth mindset is crucial to mathematics education as it helps students learn and teachers teach. Learning with the growth mindset in mathematics reflects an active learning method for acquiring mathematical knowledge, where the students position themselves to make sense of what they learn.

Therefore, this bibliometric study attempted to systematically review how the growth mindset in mathematics education has developed in recent decades so as to refresh our understanding of the gist of the literature and identify future research directions. A total of 85 studies were examined in this study. The latent topics, representative research work, development trends, collaborative organizations, and annual topic distributions will be discussed in detail.

## Methodology

Statistical bibliography is useful in revealing the development of a discipline (Pritchard, 1969). Bibliometrics uses quantitative analysis and statistics to describe pattern relationships within the research topic (Hawkins, 1981; Chen et al., 2018). Bibliometric techniques can identify current research areas and provide a roadmap for further research (Luo et al., 2022). To assess and analyze the journal impact factors of articles, the current study also processed the qualitative data in the literature. The Web of Science (WOS) and Scopus databases were used. The WOS is a broader platform for scientific information, while Scopus is a comprehensive bibliographic database that provides article abstracts and citations of peer-reviewed scientific literature. Combining the two databases is significantly beneficial for reviewing the literature (Echchakoui, 2020).

## Data collection

**FIGURE 1:**
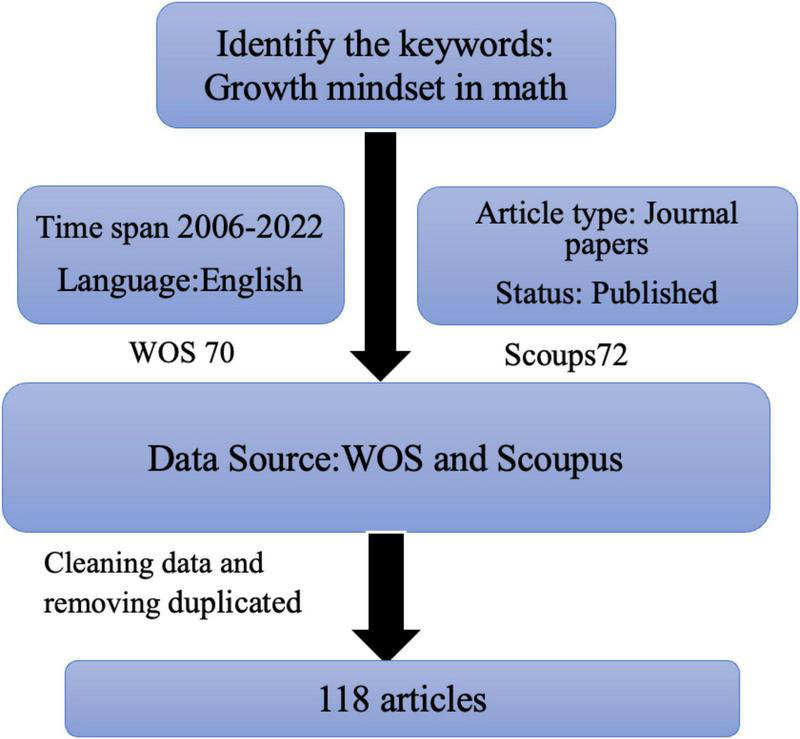
Process of data collection.

Data were collected from articles published from 2006 to 2022 retrieved from the WOS and Scopus databases. The search strings “growth mindset in math” and “growth mindset in mathematics education” were used to screen titles, abstracts, keywords, and citations to ensure relevance. After removing duplicates, the final sample comprised 85 journals and articles published from 2012 to 2022. Bibliometric analysis was conducted using techniques available in the software RStudio. The analysis reveals and corroborates several patterns in the research trends, journals, countries, and authors that have significantly impacted research on the growth mindset in mathematics education. The present dataset thus provides an updated perspective on research regarding the growth mindset in mathematics education. Figure 1 illustrates the process of data collection.

## Data analysis

The data search in the WOS and Scopus identified 85 articles relevant to the three research questions of this study. First, to determine trends in growth mindset research (RQ1), the number of articles published for each year between 2012 and 2022 was tallied and fitted on a curve. Next, the bibliometric analysis identified themes and networks among the major contributors to growth mindset research (RQ2), enabling the analysis and visualization of collaborations between researchers, as well as relationships between prolific countries/regions and institutions. Lastly, the topics of the 85 articles (RQ3) were extracted from their abstracts using the biblioshiny package for the **R** programming language (Chen et al., 2020). Structural topic modeling then enabled us to incorporate information into our model and understand how articles addressing the same topic may use different word choices in their discussions of the topic.

## Results

**FIGURE 2:**
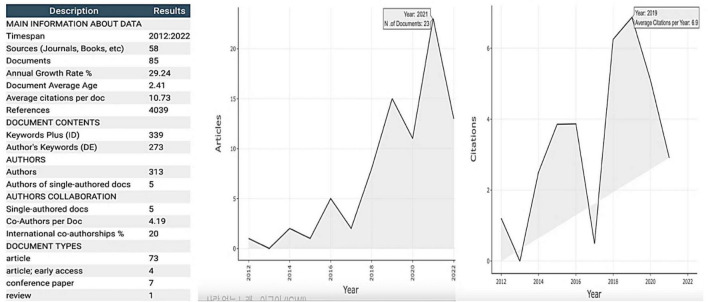
Summary statistics **(left)**, trends in the article **(middle)**, and citation **(right)** counts.

**FIGURE 3:**
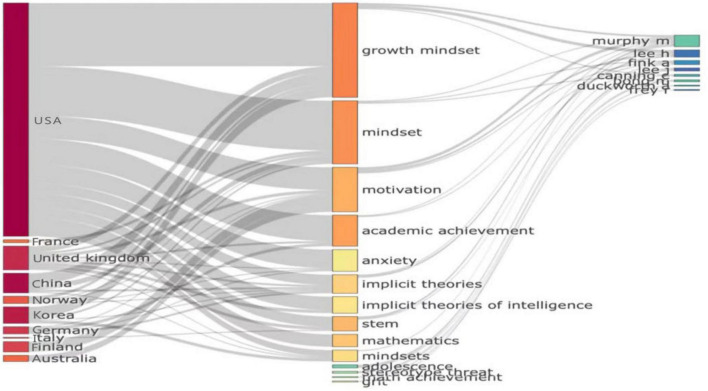
Three-field plot for countries of publications, keywords in abstracts, and authors.

Our search strings provided the flexibility for capturing various terms used to refer to the growth mindset in mathematics; however, they also yielded irrelevant search outcomes (e.g., research about STEM education) that had to be filtered out from the final sample. Figure 2 presents the results of our analysis; the table on the left displays key statistics in terms of article and citation counts, countries/regions of origin, and topics identified. The line graph in the middle illustrates the annual count for relevant articles on growth mindset research. It shows that significantly fewer articles related to the growth mindset in mathematics education had been published before 2012; from that year on, academic interest increased as the research topic evolved. Our findings also reveal that the USA, the UK, and Norway were the most productive countries in generating research on the growth mindset in mathematics education (see Figure 3). Based on the results of the analyses of themes and keywords, our findings uncover more detail about how to describe the growth mindset in mathematics education. In sum, our findings provide a representative overview of the growth mindset studies in mathematics education, offering valuable research insights for academics and practitioners looking to explore the growth mindset in the future.

### Basic summary statistics

Bibliometric indicators were employed to summarize the dataset. The present bibliographic collection includes 85 articles from 2012 to 2022. The majority were journaled articles, early access publications, and conference papers, with only one review paper in the dataset. Regarding the sources for the articles, the table in Figure 2 shows that 58 periodicals and books were represented. The table also shows that the annual growth rate for the number of articles was 29.24%, with 313 authors represented in the given time span. Turning to the keyword data, the number of author keywords (DE) was 273 words, while the number for the Keywords Plus indicator (ID) was 339. The larger number for ID compared to DE was expected because the former is a more broadly descriptive metric. Lastly, the average number of co-authors per document was 4.19, and the proportion of international co-authorships was 20%.

The line graph in the middle of Figure 2 depicts the relationship between article publications and year, which illustrates a rising trend. The year 2021 was the year with the most articles published, numbering 23. A similar rising trend is observed for the annual citation count illustrated in the line graph on the right, with 2019 having the highest number of average citations.

### Factors relevant to country of origin

To better understand the relationships between the country of origin of the documents, the keywords included in their abstracts, and their authors, we created the three-field plot (a type of Sankey diagram) illustrated in Figure 3. The plot indicates the relationships between the top countries and keywords identified among the datasets. The left column ranks the top 10 countries in published articles, namely, the USA, France, the UK, China, Norway, Korea, Germany, Italy, Finland, and Australia. The middle yellow column ranks the top 14 keywords, namely, “growth mindset,” “mindset,” “motivation,” “academic achievement,” “anxiety,” “implicit theories,” “implicit theories of intelligence,” “STEM,” “mathematics,” “mindsets,” “adolescence,” “stereotype threat,” “mathematics achievement,” and “grit.” Lastly, the right column ranks the top authors according to the number of published articles they have.

The plot shows that articles from Finland and Australia included “motivation” as a keyword, while articles from Korea, China, and Norway included “growth mindset” as a keyword. Articles from the USA targeted the broadest set of keywords, covering 9 out of 14 terms. Finally, the top authors represented in the plot include Murphy, H. Lee, Fink, J. Lee, Canning, Bong, Duckworth, and Frey.

**FIGURE 4:**
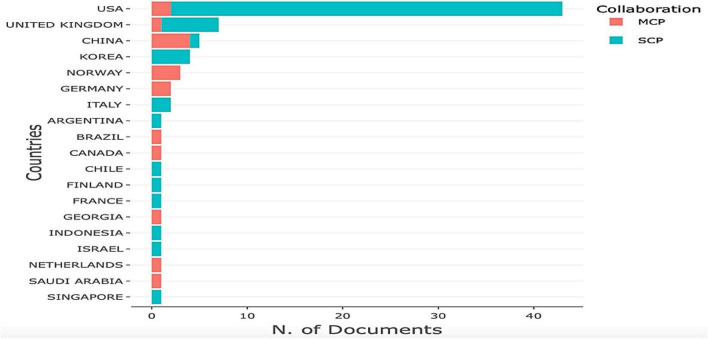
Multi-country (MCPs) and single-country (SCPs) publications by country.

Figure 4 illustrates the distribution of multi-country publications (M) and single-country publications (SCPs) in the dataset by country; MCPs refer to articles with at least one co-author representing a different country than that of the corresponding author. The bar chart shows that the top four countries represented in MCPs are China, Norway, USA, and Germany, while the top four countries represented in SCPs are USA, UK, Korea, and Italy.

**FIGURE 5:**
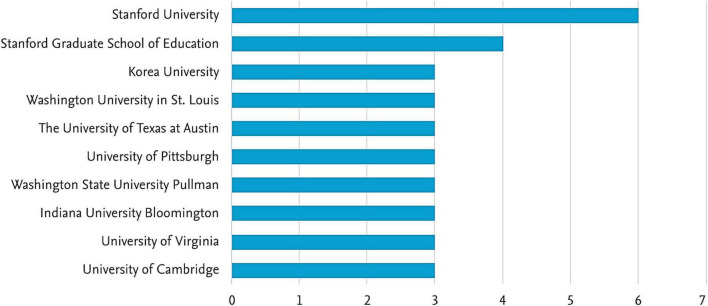
Top 10 most prolific institutions.

Figure 5 illustrates the top 10 most prolific institutions publishing articles on growth mindset research in mathematics from 2012 to 2020, of which 9 are in the US and 1 is in Korea. The top three institutions are Stanford University, Stanford Graduate School of Education, and Korea University.

### Sources and authors

**FIGURE 6:**
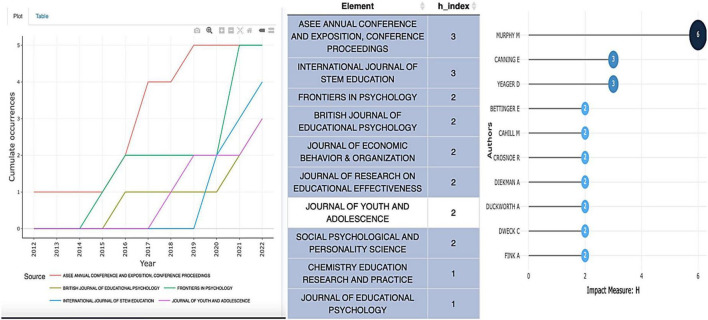
Relationships between authors, publication sources, and *h*-index.

In Figure 6, the frequency plot on the left shows the cumulative occurrences of the top five publication sources represented in the dataset. Occurrences generally increased between 2012 and 2022, with individual sources displaying different degrees of fluctuation. The most impactful source was the annual conference proceedings for the American Society for Engineering Education (*ASEE Annu. Conf. Expo.*), followed by four journals including *British Journal of Educational Psychology* (*Br. J. Educ. Psychol.*), *Frontiers in Psychology* (*Front. Psychol.*), *International Journal of STEM Education* (*Int. J. STEM Educ.*), and *Journal of Youth and Adolescence* (*J. Youth Adolesc.*). The frequency plot offers one indicator for the impact of the sources over time by illustrating periods of increase and plateauing. For instance, the number of documents published in *Front. Psychol.* and *J. Youth Adolesc.* remained at two during the years 2016–2020 and 2019–2021, respectively. Similarly, the number for *Br. J. Educ. Psychol.* stagnated at only one document during the years 2016–2020.

Another indicator for discovering the impact of sources is the *h*-index (Hirsch index), which measures the number of published articles (*h*) by an author or journal that have been cited at least *h* times (Aria and Cuccurullo, 2017). The table in the middle of Figure 6 shows *ASEE Annu. Conf. Expo.* and *Int. J. STEM Educ.* to be the top two sources, each with an *h*-index of 3. Notably, the two journals with an *h*-index of 1—*Chemistry Education Research and Practice* and *Journal of Educational Psychology*—include articles published by the top three most impactful authors by *h*-index listed in the graph on the right of Figure 6, namely, Murry (6), Canning (3), and Yeager (3).

### Keyword and topic distributions

Among the different groups of significant terms related to publications, keywords indicate essential concepts found in the abstract and main text of articles while also functioning as search terms that help readers easily find key themes, providing more information to guide data searches. Words included in an abstract provide an overview of the content of the manuscript and a guide to its essential written components, capturing the core of an article. Lastly, the titles themselves convey the principal topics of studies and highlight the significance of their findings to attract readers (Chen et al., 2018). Consequently, identifying author keywords, associated phrases, and important terms in titles and abstracts is essential for understanding the essence of a group of articles.

**FIGURE 7:**
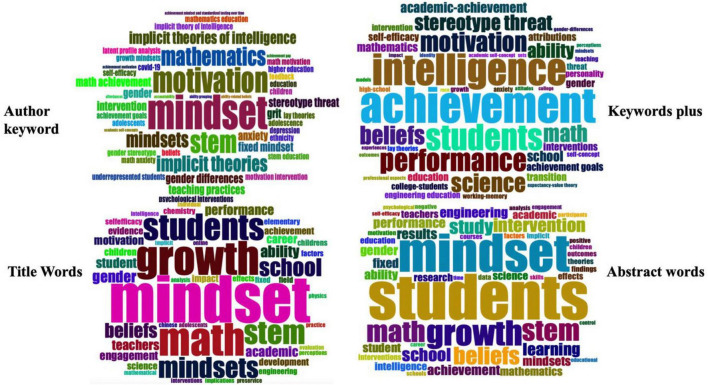
Clockwise from **(top left)**: word clouds representing author keywords, “Keywords Plus” phrases, words in abstracts, and words in titles.

Figure 7 illustrates word clouds generated using the bibliometric package in RStudio, with larger-sized words representing terms that appeared more often across articles. In the author keyword cloud, the top words are “mathematics,” “motivation,” “mindset,” “STEM,” and “implicit theories.” Additional important terms in the “Keywords Plus” cloud include “stereotype threat,” “intelligence,” “achievement,” “students,” “beliefs,” “performance,” and “science.” Lastly, other important terms found in the title and abstract word clouds include “growth”, “intervention”, “learning”, and “math”. The thematic connections among these words may represent latent trends in research on the growth mindset (Figure 3).

**FIGURE 8:**
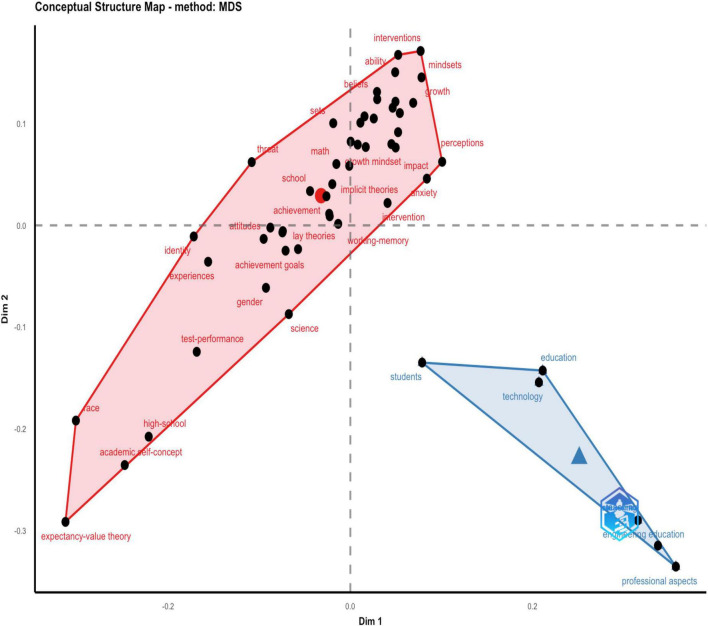
The conceptual structure of factor analysis.

While word clouds are effective in visualizing keywords, they are insufficient for understanding the connection between these important terms and the topics they address. Figure 8 presents a conceptual map that attempts to depict the connections between concepts and ideas using multidimensional scaling (MDS). In the map, words that have similar distributions along the two dimensions appear closer together (Aria and Cuccurullo, 2017). This map depicts the average position of all column profiles. The distributions of keywords on the map illustrate the two core topics of mathematics and education, with keyword clusters related to the topics falling within the respective polygons that represent them, namely, pink for mathematics and blue for education. The size of the polygons and the number of keyword points within them show that mathematics is the more broadly addressed topic, including 53 keywords such as “mindset conception,” “impact,” “test performance,” “growth mindset,” and “ability.” Conversely, the blue triangle representing education only includes six keywords: “education,” “professional aspects,” “students,” “engineering education,” “teaching,” and “technology”.

**FIGURE 9:**
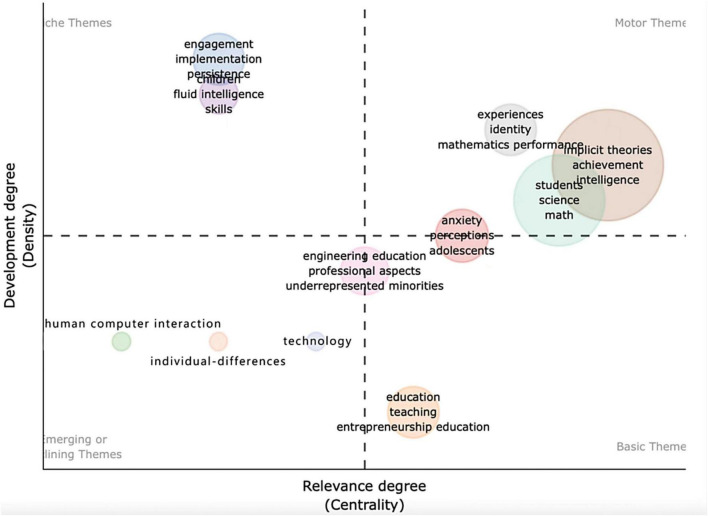
Bubble chart mapping thematic networks.

To further understand the themes addressed by the topics and keywords included in the articles, mixed methods thematic network analysis can be conducted to clarify the relationships between concepts and terms. It has been discovered that a single broad overarching thread deriving from a growth mindset connects further to the keyword, thereby establishing a thematic link between the nature of the study where the research is being carried out. Figure 9 presents a bubble chart generated to visualize the various themes addressed by the articles. The distances of thematic network bubbles from the central axes are functions of their relevance to growth mindset research in mathematics education and their degree of development. The chart shows that thematic networks are distributed across all four quadrants. Highly developed and isolated themes in the upper-left quadrant include engagement, implementation, persistence, children, fluid intelligence, and skills. Declining themes in the lower-left quadrant include computers, individual differences, and technology, while emerging themes in the same quadrant include adolescents, stereotype threats, and academic achievement. Lastly, the basic transversal themes in the lower-right quadrant include education, teaching, and entrepreneurship education. Only a few thematic bubbles straddle two quadrants, such as engineering education, professional aspects, and underrepresented minorities in the bottom quadrants. This indicates that there are still a few topic contents related to the growth mindset.

**FIGURE 10:**
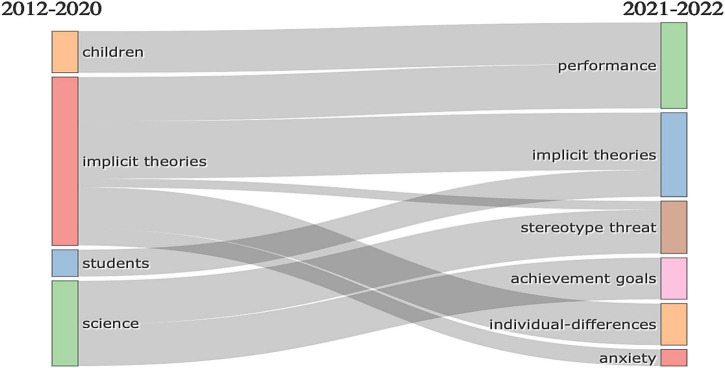
Flow diagram of longitudinal thematic evolution.

In the graph showing the distribution and development of research topics over time, a shift over the ten years can be noticed (see Figure 10). While topics in a given research field may have something in common, they can also branch out into other areas of study. Building on the resurgent interest in mapping scientific paradigms (e.g., using a flow structure), Figure 10 presents a flow diagram indicating the longitudinal evolution of research themes included in the dataset. The left side lists the top keywords in growth mindset research during 2012–2020, namely, “children,” “implicit theories,” “students,” and “science.” The right side lists the top keywords for 2021–2022, namely, “performance,” “implicit theories,” “stereotype threat,” “achievement goals,” “individual differences,” and “anxiety.” The flows that connect the two lists of keywords illustrate an evolution in the research from the concept of mindsets to implicit theories with the growth mindset alongside academic achievement. Notably, “implicit theories” has branched out into “individual differences” and “anxiety”, while “students” have fed back into “implicit theories.” This visualization of keyword trends offers more explicit details about the evolution of growth mindset research, specifically illustrating the merger of “implicit theories” and “students”.

## Discussion

This section discusses the results of our research and introduces the potential implications for future practice and research.

### Growth mindset research in different countries

As regards the results of factors relevant to the country of origin (section “Factors relevant to country of origin”), they are used to visualize the structure, description, and monitoring of published research in a particular field (Garfield et al., 1964). Figure 3 depicts a three-field plot hosting the corresponding author’s country, the name of the authors, and keywords that define the central theme of the article. Figures 4, 5 refer to the institutions wherein the authors are either employed or are professionally associated to undertake the research in their respective field of study. They all unveil several factors relevant to growth mindset research in different countries by examining keywords, institutions, and sources. Describing and analyzing the topic of the growth mindset from an empirical perspective, we surveyed important bibliometric factors related to countries, sources, authors, and keywords. We also identified associations between the emergence of growth mindset research and several important keywords such as motivation, intelligence, and performance. Our findings indicate that the USA has the most articles discussing the growth mindset. The reason is that the concept originated in this country. Dweck (1999) made a very substantial contribution when she introduced the idea of a growth mindset in 1999. The definition has since been refined and added to. By 2015, Dweck proposed that competencies can be developed through dedication and hard work, and it is this perspective that creates the love of learning and the resilience that is essential to outstanding achievement (Dweck, 2015). Articles from the USA also targeted the broadest set of top keywords compared to articles from other countries. However, it is also noted that Korea, China, and Singapore have also made substantial contributions (see Figures 3, 4). This is an encouraging sign that not only Western but also Asian academics are gradually developing and investigating the growth mindset in their own educational systems. However, does the study conducted in each country have a unique interpretation of the development mindset? Are there any differences or special features in the development of a mathematical growth mindset across different regions? The answers to these questions deserve further deep exploration.

Considering the concept of the growth mindset, which originated in the USA, Western researchers have developed a deeper conceptual understanding of the topic and produced more research output than others. Understanding the influence of the growth mindset, particularly the intervention of changing the fixed mindset, is meaningful because it may be used in countries with diverse student populations, such as the USA—combatting stereotype threats successfully could have a significant impact on student achievement (Altbach, 2004). Around the world, nations are becoming more and more diverse and multicultural, and immigrants may enter nations via land, sea, and air. Regarding the complexity of cultural context for different students, a culture-specific growth mindset (e.g., Zeeb et al., 2020) deserves future research attention. Moreover, the focus on mathematics itself is a culturally transmitted body of knowledge (Stigler and Baranes, 1988). For instance, the system of Hindu–Arabic numerals that we typically use to represent numbers is applied in the majority of the world, particularly in schools, thus promoting a level of commonality across cultures in mathematical knowledge. Nonetheless, if we combine a growth mindset with different cultures of mathematics learning, does this concept show commonality? What will transpire? Will this be modified or accepted? At this point, it is worthwhile to investigate the applicability of growth mindset theory to other geographic regions, such as Asia, which requires further exploration that considers different cultural backgrounds and beliefs about mathematics learning. Nelson et al. (1993) emphasized the importance of culture in the classroom for the mental health of students living in multicultural neighborhoods. They gave examples of some mathematics topics, showing different approaches to these topics developed in different cultures. Students have a chance to appreciate that there are many different ways of arriving at the same answer. In this setting, the use of multiculturalism is important and necessary in this context in order to foster better self-development and intercultural understanding. Therefore, future research should consider more diverse cultural backgrounds and perceptions of growth mindsets in mathematics learning.

### Growth mindset research in students’ learning

Our findings identified four groups of important terms in the dataset, namely, author keywords, “Keyword Plus” phrases, title words, and abstract words. Excluding search terms, the most frequent keywords were “motivation”, “STEM”, “implicit theories”, “intelligence”, “achievement”, “beliefs”, “anxiety”, and “intervention”. In conclusion, mathematical mindsets or implicit theories (“implicit theories”) include students’ self-concept and self-efficacy beliefs (“beliefs), as well as their attitude about failure, which includes math anxiety (“anxiety”), all of which determine their willingness to learn mathematics (Meece et al., 1990; Panaoura et al., 2009). Additionally, students’ mathematical beliefs are often cyclically related to their academic achievement (“achievement”); in turn, positive feedback on academic achievement provides students with positive motivation to learn (Ross et al., 2012). Therefore, students’ academic achievement (“achievement”) and motivation (“motivation”) are closely related to the growth mindset (Zhao et al., 2018). On the other hand, a growth mindset sees intelligence (“intelligence”) as a moldable trait that is not fixed and could be improved through effort and intervention (“intervention”) (Macnamara and Rupani, 2017; Zhu et al., 2019). Additionally, the theories and intervention methods related to mathematical beliefs could also be applied to other STEM-related (“STEM”) disciplines (Boaler et al., 2021).

When we examine each keyword thoroughly, it is not difficult to conclude that there are insufficient growth-mindset-related keywords. The available analysis is very simplistic, and no additional keywords interacted. Perhaps the lack of growth-mindset-related research publications is the reason for this study constraint. However, if we examine the field of mathematics education, it is widely considered that the addition of the growth mindset theory to future study will yield greater opportunities. Instead of just examining and comprehending this theory, we expect that in the future, the direction and content of the analysis will be clarified.

Moving on to focusing on the keywords and determining their commonality between the research areas, the conceptual structure map, as represented in Figure 8, provides a high-level overview of the keyword clusters analyzed from the bibliometric information. Two clusters were formed using multivariate correspondence analysis by determining their commonality. Surprisingly, segment topics that have gained traction are “academic self-concept”, “test-performance”, “lay theories”, and “expectancy-value theory”. It seems that researchers are attempting to explain and describe the growth mindset through various theories and students’ academic performance. Regarding the studies by by Sarrasin et al. (2018) and Yeager et al. (2019), the growth mindset had positive effects on student motivation and academic performance. However, the findings of the keyword analysis in growth mindset revealed no topics related to specific subfields in mathematics, such as algebra, calculus, or geometry. Learning mathematics should help students acquire knowledge and skills in arithmetic, algebra, geometry, statistics, space, and structure logically and systematically (Vinner, 2002). As for the teaching of mathematics, different parts of mathematics may require different pedagogies to inspire students’ mathematical thinking. Hence, researchers may need to incorporate mathematical content in the field of growth mindset research in the future, to foster the growth mindset among students in mathematics learning. The previous literature review indicated that learning with a growth mindset could be considered a very promising approach for students in learning mathematics. However, in the results of the keyword analysis, we did not see any keywords related to students’ learning, such as classroom learning or classroom activity.

Another gap in the research of the analysis of the keywords was that it did not address the topic of teachers’ mindsets and parents’ mindsets. The influence of teachers and parents on students’ mathematics learning has been well-investigated in the literature. It seems necessary to investigate the impact of teachers’ mindsets or parents’ mindsets on students’ growth mindsets as well. Analysis in such research could be applied to the teacher’s mindset of their past learning experience and associated with teaching practices. For instance, if a teacher does not have a growth mindset in mathematics teaching and learning, can the teacher still teach mathematics effectively? Can a teacher’s mindset be changed when the teacher wants to change their students’ fixed mindset? What would be the differences between teachers with and without a growth mindset, when they aim to promote their students’ growth mindset? Few studies mentioned the relationship between the characteristics of mathematics teachers and their growth mindset (Shoshani, 2021). Those questions related to teachers are possible inquiries for further study.

To conclude, research associated with more keywords needs to be discussed, including comparative studies of mathematics learning at different grade levels, student learning habits, self-regulation, self-efficacy, mathematical thinking, and even teachers’ mindsets. In addition to considering the topic matter itself, researchers should also consider the learning experiences of students. The development of a growth mindset that enables learners to focus their attention is necessary for learners to raise their interest and motivation in mathematics learning; these strategies should be discussed in future research. Finally, in practical terms, future educators should investigate how to develop growth mindsets and skills progressively among their students, implementing their insights into teaching designs.

### Growth mindset research in different themes

When facing the future challenges of a complex and uncertain world, school education is undergoing a competency-based curriculum reform (Organisation for Economic Co-operation and Development [OECD], 2018). Students who are best prepared for the future are change agents. They need a broad set of knowledge, skills, attitudes, and values in action, including broad and specialized knowledge, cognitive and meta-cognitive skills, social and emotional skills, and practical and physical skills. The use of this broader range of knowledge and skills will be mediated by attitudes and values (Organisation for Economic Co-operation and Development [OECD], 2018). To process and apply their knowledge and skills in unknown and evolving circumstances, mathematics can play a crucial role. Mathematics and its applications permeate lots of facets of contemporary life. However, mathematics, the significance of which we feel affects every area of our lives, is not sufficiently learned by many people for various reasons. This might be due to the methods and tactics for learning mathematics, and also may relate to students’ mathematics learning difficulty (MLD) (Von Aster and Shalev, 2007; Hartmann, 2013), or mathematics anxiety (Huang et al., 2019; Samuel and Warner, 2021). This may further explain why the thematic evolution in Figure 10 is trending toward concretization, from student themes to an emphasis on student anxiety and individual differences in mathematics learning, for example. However, if we further examine the thematic network, it is evident that various themes are involved, and their connections appear to be very scattered and macroscopic, such as gender and intelligence. Obviously, it is evident that those studies are still shallow (as in Figures 8, 9). Surprisingly, Figure 9 involves two variables: entrepreneurial education and technology. It can be concluded that even though the concept of the growth mindset is being investigated in depth, the related connections still need to be explored more actively. Such as how to use this concept of a growth mindset sufficiently to link technology to game-based learning in the classroom. Can entrepreneurial education contribute to the growth of students’ leadership capabilities? These associations are not seen in the figure, and these factors are not adequately studied in the mathematics discipline. No factors such as the growth mindset versus geometry or algebra were addressed in mathematics. Researchers are still regarding the discipline of mathematics as a whole, and lack consideration of different learning areas in mathematics, which may make a difference in the development of a mathematical mindset. Some specific questions can be further explored, such as how can growth mindsets can be used to facilitate student learning in the domain of numbers.

## Conclusion

This review focused on research about the growth mindset in mathematics, drawing on article metadata from two different databases. Since researchers have begun exploring the growth mindset in mathematics education, an increasing trend in research outputs has been observed since 2012, with achievement and academic success becoming popular topics in education research. Given that no prior study has used quantitative analysis and statistics to investigate pattern relationships in the field of the growth mindset as a research topic, the current study adopted the bibliometric package in RStudio to analyze 85 studies published from 2012 to 2022, revealing notable trends and hidden relationships in growth mindset research.

The findings of this study address prevalent subject areas and find new networks of research topics for the growth mindset in mathematics education. They may help mathematics educators gain a deeper, more diverse understanding of current research on the theme, which can then help them design or explore possible effective strategies for the development of students’ growth mindset. However, we acknowledge that this review remains limited as it only analyzed limited journal articles published within the past decade. Different types of documents such as research reports or book chapters from more databases can be considered. Nevertheless, based on the results of this review, we make several recommendations for future practice and research. Our findings suggest that additional aspects should be considered in research on the growth mindset in mathematics teaching and learning, including mathematical knowledge, cultural differences, and learner characteristics. To sum up, this review contributes to the understanding of the primary topics in the research on the growth mindset in mathematics, the concept of the growth mindset, and possible directions for further research on the growth mindset in mathematics education.

## Author contributions

XX, QZ, and JS: conceptualization, writing, review, and editing the manuscript. XX, YW, and QZ: methodology. XX and YW: formal analysis. XX and QZ: writing the manuscript. All authors have read and agreed to the published version of the manuscript.
